# Benefits and challenges of using the cohort multiple randomised controlled trial design for testing an intervention for depression

**DOI:** 10.1186/s13063-017-2059-4

**Published:** 2017-07-06

**Authors:** Petter Viksveen, Clare Relton, Jon Nicholl

**Affiliations:** 1 The Department of Health Studies, University of Stavanger, Kjell Arholms hus, Kjell Arholms gate 39, 4021 Stavanger, Norway; 2 School of Health and Related Research, The University of Sheffield, Regent Court, 30 Regent Street, Sheffield, S1 4DA UK

**Keywords:** Pragmatic trials, Cohort multiple RCT, Recruitment, Depression, Trials within cohorts

## Abstract

**Background:**

Trials which test the effectiveness of interventions compared with the status quo frequently encounter challenges. The cohort multiple randomised controlled trial (cmRCT) design is an innovative approach to the design and conduct of pragmatic trials which seeks to address some of these challenges.

**Main text:**

In this article, we report our experiences with the first completed randomised controlled trial (RCT) using the cmRCT design. This trial—the Depression in South Yorkshire (DEPSY) trial—involved comparison of treatment as usual (TAU) with TAU plus the offer of an intervention for people with self-reported long-term moderate to severe depression. In the trial, we used an existing large population-based cohort: the Yorkshire Health Study. We discuss our experiences with recruitment, attrition, crossover, data analysis, generalisability of results, and cost. The main challenges in using the cmRCT design were the high crossover to the control group and the lower questionnaire response rate among patients who refused the offer of treatment. However, the design did help facilitate efficient and complete recruitment of the trial population as well as analysable data that were generalisable to the population of interest. Attrition rates were also smaller than those reported in other depression trials.

**Conclusion:**

This first completed full trial using the cmRCT design testing an intervention for self-reported depression was associated with a number of important benefits. Further research is required to compare the acceptability and cost effectiveness of standard pragmatic RCT design with the cmRCT design.

**Trial registration:**

ISRCTN registry: ISRCTN02484593. Registered on 7 Jan 2013.

## Background

Since its publication in 2010, a number of studies have started using the innovative cohort multiple randomised controlled trial (cmRCT) design [1], also known as ‘Trials within Cohorts’ (www.twics.global). The cmRCT design uses a large observational cohort of people with the condition of interest, with regular measurement of outcomes for the whole cohort. This cohort provides a capacity for multiple randomised controlled trials (RCTs) over time. For each RCT, routine cohort data help to identify those eligible for the trial. Eligible participants are then randomly selected to be offered the trial intervention, and their outcomes are compared with those not randomly selected (i.e., those receiving usual care). Like some cluster trial designs, the cmRCT design uses a ‘randomisation without prior consent’ approach to informed consent [2]; thus, information about the intervention is provided only to the intervention group, and this information is given after (not before) randomisation.

Researchers in a variety of settings (including hospital, primary care and community) in the United Kingdom, Canada and The Netherlands use the design [3], including a number of studies testing interventions for mental health in the United Kingdom [4, 5] and Canada [6]. In this article, we report on our experiences of using the cmRCT design in relation to recruitment, attrition, crossover, data analysis, cost and generalisability of results for the first completed full-scale RCT of the cmRCT design: the Depression in South Yorkshire (DEPSY) trial. In this trial, we used an existing cohort to compare treatment as usual (TAU) with TAU plus the offer of a course of treatment provided by a homeopath for patients with self-reported long-term moderate to severe depression.

## Main text

### Recruitment

Trials often struggle to reach recruitment goals on time, and many trials fail entirely to recruit a sufficient number of participants [7], especially trials in depression [8]. This is also true for pragmatic trials of interventions such as counselling and cognitive behavioural therapy for depression [9–11]. Consequences of insufficient recruitment include at best reduced power and/or extended recruitment periods, contributing to increased costs, and at worst inability to draw conclusions because of underpowered trials and wasted resources.

Recruitment to the DEPSY trial was from an established population-based cohort (the Yorkshire Health Study; www.yorkshirehealthstudy.org [12]) set up to facilitate multiple pragmatic trials using the cmRCT design. All people in the cohort had previously provided self-reported information on their health (including anxiety or depression), health-related behaviours and health care resource use, and had given consent to be contacted again. The researchers invited those who had reported long-term depression or feeling moderately or severely anxious or depressed when completing the Yorkshire Health Study questionnaire (*n* = 5740), to complete a detailed mood and health questionnaire. Completed questionnaires were returned by 2214 patients (38.6%). This provided the trial researchers with the information needed to apply the inclusion/exclusion criteria, as well as baseline information for the trial. All cohort patients had given permission for their data to be used to look at the benefit of health treatments and to be contacted again by the researchers. Additionally, written informed consent was requested by patients taking up the offer of treatment.

It took a total of 5 months for 566 eligible participants (17% more than the original sample size calculation) to be recruited to the DEPSY trial [13]. The method used avoided many recruitment barriers encountered in other trials, where clinicians may struggle to find time to recruit patients, feel reluctant to let the research disrupt consultations, or consider the randomisation process to be inappropriate and the research process to be too much of a burden for patients [8–10, 14].

The majority of people take part in trials in the hope that they will obtain direct and/or indirect benefits for themselves or others as a result of their participation (e.g., improved health) [15]. It is common for patients to refuse randomisation because of the risk of not being offered the treatment of their preference [8, 16]. The use of the cmRCT design avoided this barrier because only those randomly selected to receive an offer of treatment were informed about the intervention being tested [1]. All 185 patients in the ‘Offer’ group were sent a letter offering them the treatment, 150 of whom were reached by telephone. About half (*n* = 95, 51.4%) accepted the offer of treatment. Everyone in both the ‘Offer’ and ‘No offer’ groups were sent baseline and follow-up questionnaires, unless they asked not to be sent any further questionnaires (*n* = 62, 11.0%).

### Attrition

Patients who agree to participate in trials may be disappointed if they are not allocated to receive their preferred intervention [17]. As a consequence, they may become uncooperative, report poorer outcomes than experienced and even leave the trial, which may in turn significantly affect analyses and the interpretation of results [18]. Such attrition may lead to biased results and, at worst, inability to draw conclusions [19].

The fact that patients in the TAU control arm were unaware of the intervention being trialled may have contributed to lowering attrition rates (*attrition* here refers to non-completion of follow-up questionnaires at 6 and 12 months). Following discussion with other mental health researchers, it was estimated that a realistic response rate would be 60% [13]. Results showed that more than 80% returned completed questionnaires at 6 months, and 67% returned them at 12 months. When considering drop-out for treatment among those who accepted the offer of treatment in the offer group, over 90% followed their treatment with several consultations. Other researchers have found attrition rates of, for example, 16% for psychotherapy and 32% for parenting education [20]. Researchers in trials of antidepressant treatment found attrition rates of 23% at 6 months and 47% at 12 months [21].

The rate at which people completed follow-up questionnaires was lower (68%) in the group randomly selected to receive the offer of the intervention (Offer group), compared with the control group (87%), where patients were not offered the intervention (No offer group). In trials using the cmRCT design, some patients in the Offer group may be uninterested in responding to questionnaires if they either have no interest in or dislike the intervention [18]. This will not be an issue for patients in the No offer group, because they are unaware of the intervention. It could therefore explain a between group difference in questionnaire response rates.

Within the Offer group, 88% of those who accepted the offer and received treatment returned the completed questionnaire (equal to the response rate in the control group), compared with only 54% of those who did not take up the offer (non-accepters). To understand what might have contributed to these differences, baseline characteristics of the four groups (Offer and No offer group responders and non-responders at 6 and 12 months) were compared using a multiple linear regression model for each baseline covariate, as recommended by Walters [22]. There was no evidence of significant differences between the four groups in regard to their depression or anxiety scores or in any other baseline covariates considered to be likely to influence outcomes. Between-group comparisons could therefore be carried out with limited risk of significant influence of known potential confounding factors.

Although there were no known characteristics that could explain a lower return rate of questionnaires in Offer group non-accepters, the question remains why this particular group was less likely to return questionnaires. One possible explanation could be that non-accepters upon receiving follow-up questionnaires thought that their response was not needed, because they had not taken up the offer of treatment. Moreover, patients who do not believe in the intervention or who have unsuccessfully tried it in the past may not be interested in participating in the trial, which was reported by two patients in this trial. Further research is required to understand non-response in trials using the cmRCT design.

### Crossover and data analysis

Patients in pragmatic trials not randomly selected to receive their preferred intervention may seek the intervention outside the trial, thereby contributing to bias resulting from crossover [23]. Van der Velden et al. [18] suggested there is little risk of crossover from the control group when using the cmRCT design, because patients in the control group are not given information about the intervention being trialled. We collected data on patients’ use of interventions (including the treatment used in this trial) and did not find any cases of crossover from the control to the intervention arm. A bigger challenge in the cmRCT design is ‘crossover’ from the intervention to the control arm by patients not accepting the intervention being offered and remaining on TAU. In the DEPSY trial, 40% took up the offer of treatment and received treatment; thus, 60% crossed over to the control group. This has implications for the analysis of results.

Using an intention-to-treat (ITT) analysis when significant proportions cross over, results in ‘watered-down’ estimates of potentially effective interventions [24, 25]. Such crossover is to be expected in trials using the cmRCT design [18]. Per-protocol analyses are often used by researchers as an additional (or in some cases as the only) analysis, but this approach carries a risk of bias.

For the DEPSY trial, we used an ITT analysis to estimate the effect of the offer of treatment, as well as an instrumental variables analysis [26], which is a type of complier average causal effect analysis that takes into account baseline values and where randomisation is the instrument [25], to assess the effectiveness of treatment received. This analysis compares Offer group patients who have received the intervention with patients in the No offer group who would have received the intervention if they had been offered it. This analytic method has been recommended as the secondary analysis used in RCTs because it carries lower risk of bias than per-protocol or on-treatment analyses [25].

### Generalisability of results

The aim of pragmatic trials is to produce results which are generalisable to the relevant clinical population. Therefore, whenever possible, the intervention should be provided in a way that is comparable to regular practice to a trial population that is comparable to the population of interest, and the information provided (and consents sought) also should be as similar as possible. It is not common in real-world practice to give patients information about interventions that they cannot receive. Commonly, available treatment options are discussed, and treatment plans are agreed with patients. The standard procedure for RCTs is that treatment is decided by chance, and information is not tailored to the individual patient but is generic, regardless of whether the patient is offered the treatment. In trials with the cmRCT design, only those randomly selected to be offered the intervention are provided with information about the intervention. Hence, patients in the No offer group are not informed about interventions they cannot receive. This is more comparable to real-world practice and contributes therefore to increasing the generalisability of results.

The seven practitioners delivering the intervention had been instructed to practice in the normal manner. Therefore, as in everyday practice, no treatment protocols were provided for them, other than guidelines for how to deal with risk issues and adverse events. Therefore, the frequency and length of consultations, as well as the medications and advice given, were comparable to routine practice.

Analysis of the self-reported data showed that the DEPSY trial participants had several similarities to the general population of patients with depression. Depression was significantly correlated to commonly seen comorbidities (anxiety and obesity), a greater chance of being unemployed [27] and deprived [28] than the general population, and with a larger proportion of women [29] (further details in trial article). Patients more commonly had chronic depression and depression was not diagnosed but was self-reported using the Patient Health Questionnaire (PHQ-9). The PHQ-9 has been found to have a high degree of validity, reliability, sensitivity and specificity, and it is sensitive to change and useful for patients with a variety of comorbidities [30]. Though not intended to replace diagnostic interviews, it has been found to be more conservative than clinician-rated outcomes [31]. The results of the trial are in many respects therefore more likely to be generalisable to the population of patients with chronic self-reported moderate to severe depression.

### Cost

Recruitment to the trial via the Yorkshire Health Study cohort cost £15,000 (access to the cohort, researcher time, and printing and mailing out questionnaires and letters), which equates to £26.50 per participant recruited. Identifying and recruiting participants from a large cohort then enabled the DEPSY trial to use unequal randomisation (1:2 intervention to control). This meant that (compared with 1:1 randomisation) 25% fewer patients were offered the intervention, thus reducing the trial intervention costs by £10,000.

## Conclusion

The main benefits of using the cmRCT design to test an intervention for self-reported depression were full, fast and efficient recruitment; lower attrition rates than other depression trials; and a trial population broadly similar to the general population of patients self-reporting chronic moderate to severe depression. The main challenges in using the cmRCT design for this pragmatic RCT were the lower follow-up (questionnaire response) rate in those who refused the offer of the intervention and the large ‘crossover’ (60%) from the intervention to the control group. The data nevertheless allowed us to carry out an ITT analysis for the offer of treatment and also to assess the effectiveness of treatment received using an instrumental variables analysis. Further research is required to compare the acceptability and cost-effectiveness of a standard pragmatic RCT design with the cmRCT design for research in all fields of healthcare, including mental health.

## Abbreviations

cmRCT: Cohort multiple randomised controlled trial
DEPSY: Depression in South Yorkshire
ITT: Intention to treat
PHQ-9: Patient Health Questionnaire
RCT: Randomised controlled trial
TAU: Treatment as usual
